# Dimensions of antenatal care service and the alacrity of mothers towards institutional delivery in South and South East Asia

**DOI:** 10.1371/journal.pone.0181793

**Published:** 2017-07-25

**Authors:** Priyanka Dixit, Junaid Khan, Laxmi Kant Dwivedi, Amrita Gupta

**Affiliations:** 1 Tata Institute of Social Sciences, Mumbai, Maharashtra, India; 2 International Institute for Population Sciences, Mumbai, Maharashtra, India; Centre for Injury Prevention and Research, Bangladesh (CIPRB) & Örebro University, Sweden, BANGLADESH

## Abstract

**Background:**

A number of studies have assessed the effectiveness of antenatal care (ANC) on uptake of institutional delivery care. However, none address the issue of association between the different components of ANC i.e. ANC component which is independent of health care delivery systems (timing and number of ANC visits), ANC components which depends on health care delivery systems (specific ANC procedures that women receive) with institutional delivery.

**Methods:**

Data for the study has been taken from the DHS conducted in the six selected South and South-East Asian countries during 1998–2013. The two dimensions of ANC are the key predictors. The outcome variable is a binary variable, where zero '0' denotes a home delivery and one '1' denotes an institutional delivery. In addition to probit estimation biprobit estimation method has been used to correct for the possible endogeneity.

**Findings:**

Analysis suggests that both the factors show a positive effect on institutional delivery but the level of associations are different. Probit estimation for each country suggests that the association is higher for the factor- which depends on health care delivery systems than the other factor. After correction of endogeneity through biprobit estimation we get the true associations for both the dimensions and it confirms that the ANC components which depends on health care delivery systems is more associated with the utilization of institutional delivery than the other factor.

**Conclusions:**

The content of care may fulfill the women’s need and expectations while visiting for ANC care. The study suggests that the quality of antenatal care must be improved which depends on health care delivery systems to motivates the women to utilize the institutional delivery.

## Introduction

Globally the number of women who die each year from pregnancy-related complications and childbirth has declined from 532,000 in 1990 to 303,000 in 2015 [1]. Despite the significant reduction in maternal deaths, dishearteningly there exists a large disparity between developed and the developing nations. The developing countries have 14 times higher maternal mortality ratio as compared to the developed countries [2]. South Asia alone accounts for 66,000 maternal deaths a year, which is around 22% of the global total [1]. Non-institutional delivery and delays in accessing such services are responsible for nearly all of the global maternal deaths in low-income countries [3,4]. A study based on 48 Demographic and Health Surveys (DHS) found that worldwide about half of the births occurred at home. Moreover, in South and South-East Asia women from poor household had higher levels of home deliveries compared to women in Sub-Saharan Africa [5].

Different countries utilize different strategies and activities to increase facility delivery like the elimination of user fees, community-based health insurance schemes, conditional cash transfers, the establishment of birth centers, provision of incentives and birth preparedness packages [6–8]. A number of studies have examined the empirical evidence concerning the influence of demographic and socio-economic factors on facility delivery in different settings such as India, Indonesia, Bangladesh and other South-East Asian countries [9–12]. Studies have also assessed the impact of utilization of previous health services on subsequent health services [13], [14]. Moreover, some studies have also evaluated the effectiveness of antenatal care (ANC) on the uptake of institutional delivery [13], [15], [16], [17].

ANC, along with promoting the utilization of subsequent health services has a vast range of benefits for pregnant women; but its effectiveness is directly dependent on the content and quality of ANC and the availability of effective referral and obstetric care services [18]. For examining the association between ANC and the utilization of institutional delivery, researchers have taken the number of ANC visits made by a woman as a measure [19], [20]. However, only the numbers of ANC visits made is not sufficient to capture the whole process of ANC service and the motivational issues for uptake of next level of services like institutional delivery.

Full ANC which is the combination of at least eight ANC visits and procedures like- tetanus vaccination, collection urine, and blood, weight, height and blood pressure measurement, counseling on pregnancy-related complications and breastfeeding [21]. In the present study, antenatal care service is conceptualized in two dimensions. One dimension is the number of ANC visits made by the women which are independent of the health care delivery systems. The other dimension is the ANC services that the women receive in the health care institutions which depend on the health care delivery systems.

The aim of this paper is to explore the uptake of ANC care services during pregnancy, and its influence through the two specified dimensions to promote institutional delivery in the six selected South and South-East Asian countries. Previous studies have not addressed the issue of association between the two dimensions of ANC i.e. ANC component which is independent of the health care delivery systems (timing and number of ANC visits) and ANC components which depend on the health care delivery systems (specific ANC procedures that women receive) with institutional delivery. Moreover, no other studies have dealt with this particular issue in any country specific analysis. Few studies have been conducted on the association between ANC services, and institutional delivery in developing countries including India. However comparative studies based on the South and South-East Asian countries is relatively scarce. The present study attempts to fill this gap by examining the situation in the six selected countries i.e. India, Bangladesh, Nepal, Pakistan, Indonesia, and the Philippines. Even though the six neighboring South and South-East Asian countries have experienced substantial upturns in the rates of institutional delivery during the last two decades, the percentage of institutional delivery is still even below 50% in all selected countries expect the Philippines and Pakistan. However, uptake of ANC care services varies from 32 percent in Bangladesh to 88 percent in the Philippines. The study will be helpful in enlightening the program and policy makers about the component of ANC to focus on for interventions which in turn would increase the utilization of institutional deliveries and subsequently improve maternal and newborn health outcomes.

## Materials and methods

Data for the study has been taken from the DHS conducted in the six selected South and South-East Asian countries during 1998–2013. The datasets are publicly available from the DHS website [22]. The DHS survey applies multistage probability sampling to provide nationally representative samples of women in the reproductive age group (15–49 years). The analysis is based on the most recent birth. To bring out the recent scenario in the uptake of maternal health care utilization in the selected countries, the DHS conducted after the year 2000 is used for the study. Table 1 presents the list of the selected countries along with years of the DHS survey that have been used for the study.

**Table 1:** DHS surveys included in the study.

Country	Survey Years	
India	1998–99	2005–06
Nepal	2006	2011
Pakistan	2006–07	2012–13
Bangladesh	2004	2007
Indonesia	2007	2012
Philippines	2003	2008

The independent variable for the study is ANC which is considered through two dimensions. The first dimension of ANC includes the number of ANC visits and the second dimension includes the specific health care check-ups that women receive during the ANC giving procedure. Place of delivery is the response variable grouped into different categories based on the place where the birth took place. Place of delivery is recoded into a binary variable, where ‘0’ denotes home delivery and ‘1’ denotes institutional delivery. The factors which describe the socio-economic and demographic characteristic of woman, as well as the independent predictors of the outcome of interest, have been controlled to check the original effect of the two dimensions of ANC on the place of delivery.

Socioeconomic and demographic predictors such as the place of residence, parental education, mother’s age at birth, mother’s working status, birth order of the last child, children ever born (CEB), decision on health care, wealth quintile, media exposure (newspaper, radio, television), religion, caste are included in the study. The independent predictors of institutional delivery include mainly the user related indicators of service access, socioeconomic and demographic factors. The place of residence is taken as a proxy to control for the differing levels of service access between urban and rural areas. The importance of age of mothers in health behavior has long been recognized. It has been suggested that younger women tend to use healthcare services more frequently than older women because the availability of modern health-care services has increased in the recent years [23]. Mother's age at childbirth was categorized into four categories- less 18 years, 19–24 years, 25–29 years and 30 and above years. The educational level of the women and their husbands provide awareness about mothers as well as child health conditions helping in the treatment seeking behavior [24]. The years of schooling is grouped into four categories- no education, primary education, secondary education and higher education. The employment status of women is grouped into two categories- employed and unemployed. As the majority of the women in study countries are not gainfully employed and add no cash to their household income, and those work, some of them work for cash and others for kind [25]. The birth order of the last child and CEB are also included to capture women’s previous experience with pregnancy and childbirth, and also the effects of family-size on health-service utilization [26]. CEB is categorized into four categories- 1, 2–3, 4–5 and 6 and above. Similarly, the birth order variable is also categorized into four categories- 1, 2–3, 4–5 and 6 and above. Wealth quintile which determines the financial well-being of the women is used to control for the economic status of the women. Wealth quintile is based on a relative index of household wealth which includes ownership of consumer items and dwelling characteristics. Households are ranked by their household scores and divided into different quintiles, i.e. poorest, poor, middle, rich and richest. Reading newspaper, listening to radio and watching television are chosen as a measure of access and communication to health messages [27]. Each of these variables is categorized into two categories- no exposure and at least once in a week. As the cultures, traditional beliefs, and norms do vary by religion and caste theses these two variables are included in the study. Religion is grouped as Hindu, Muslim and others (Sikh, Christian, Buddhist and others). Caste is grouped as Scheduled Castes (SCs), Scheduled Tribes (STs), Other Backward Classes (OBCs) and Others (general).

Based on the information available in DHS surveys for ANC services two dimensions of ANC are constructed. The first dimension of ANC includes the number of ANC visits (less than three versus three or more visits) made by women, and this dimension is independent of the health care delivery systems. The second dimension which is dependent on the health care delivery systems includes the following- whether a woman received tetanus vaccine during her pregnancy (yes versus no); whether a woman receives the following ANC procedures- measurement of weight, height and blood pressure, urine sample and blood samples were taken, breastfeeding counseling, and informed about signs of complications (yes versus no, 7 items). The first dimension includes the number of ANC visits during the total duration of pregnancy, whereas for the second dimension principal component analysis has been used to create the scores to measure the level of ANC care services. The internal reliability coefficient of these two constructs ranges from 0.56 (lowest) for Indonesia (DHS-2012) to 0.87 (highest) for India according to National Family Health Survey (NFHS) -2005-06.

For examining the empirical association, multiple regression analysis has been used. The idea behind multiple regression analysis is that if all the variables associated with both the treatment and the outcome can be included as covariates in the model, the influence of covariates can be controlled, and the remaining measure of the effect of the treatment on the outcome will be independent of the effects of any confounding factors [28]. In this study probit estimation method has been employed to examine the impact of the two dimensions of ANC on institutional delivery using two different equations as in Eqs (1A) and (1B) below.

The outcome of interest is a dichotomous variable denoting the place of delivery can be modeled as probit with independent regressors D _i1_ and D _i2_ and with the covariates Xi. Here D _i1_ and D _i2_ are Boolean variables denoting ANC components which are independent and dependent on health care delivery systems, respectively.

The variables are as follows-

D_i1_ = 1 if number of ANC visits ≥ 3

         = 0 otherwise

D_i2_ = 1 if ANC intensity score is highest

         = 0 otherwise

$$Y_{i}=D_{i1}.\delta +X_{i1}.\beta_{1}+\varepsilon_{i1}$$ (1A)

$$Y_{i}=D_{i2}.\delta ´+X_{i1}.\beta_{1}´+\varepsilon_{i1}´$$ (1B)

The error terms are assumed to be independently and identically distributed (iid) from a normal distribution with mean zero and variance one.

In addition to the probit estimation, biprobit estimation method has been used to correct for the possible endogeneity in the structure with the help of instrumental variable following exclusion criteria. According to exclusion criteria, there should be at least one instrument which affects the exposure variable but not the outcome variable. The ‘timing of the first antenatal visit’ by the women has been designed as an instrument to check the endogeneity between the exposure (ANC care) and outcome variable (place of delivery of recent birth). The strength of the biprobit method is that it allows for a test of the endogeneity of the exposure variable in the outcome equation—that is, it estimates the correlation between the unobservables affecting the outcome variable and the exposure variable. The Biprobit model is as follows-

$$O_{i1}=E_{i1}+X_{i1}.\beta_{1}+\varepsilon_{i1}$$

$$E_{i1}=IV+X_{i1}.\beta_{1}´+\varepsilon_{i1}´$$ (2)

Where “IV” is the instrumental variable, “E” is the exposure variable and “O” is the outcome variable.

## Results

### Current use of antenatal care

Table 2 shows the number of antenatal care visits that the women had for their most recent live birth in the five years preceding the DHS survey in the six selected countries. Countries like Indonesia and Philippines have relatively high level of ANC coverage as compared to the other study countries. Among the selected countries Indonesia has the highest proportion of women having more than three ANC visits while it is lowest for Bangladesh. The DHS survey conducted in Indonesia (2012) and Philippines (2008) shows that more than 85% women had more than three ANC visits and thus these two countries are considered as the high ANC coverage countries in the study. According to India’s NFHS (2005–06), the percentage of women having more than three ANC visits is 53% whereas the DHS survey conducted in Nepal (2011) shows the percentage is 67%. These two countries with more than 50% of women of having more than three ANC visits are considered as medium ANC coverage countries. The DHS survey for Bangladesh (2007) and Pakistan (2012–13), shows that women having more than three ANC visits are 31% and 49% respectively, and thus these two countries are considered as the low ANC coverage countries.

**Table 2:** Percentage distributions of three or more ANC visits, ANC visits at 1^st^ trimester and institutional delivery for the study countries.

Country/ Year	ANC visit	Total	1^st^ ANC	Total	Institutional	Total
	3+ visits		1^st^ trimester		delivery	
Bangladesh-2004	27.1	5,416	19.5	5,416	10.9	5,416
Bangladesh-2007	31.5	4,905	24.3	4,905	16.4	4,905
Pakistan-2006-07	40.1	5,677	30.6	5,677	37.0	5,677
Pakistan-2012-13	49.0	7,447	42.4	7,447	51.6	7,447
Nepal-2006	49.3	4,066	27.7	4,066	19.4	4,066
Nepal-2011	66.5	4,148	49.7	4,148	38.5	4,148
India-1998-99	44.2	28,779	33.0	28,779	33.9	28,779
India-2005-06	52.8	39,677	43.9	39,677	41.7	39,677
Indonesia-2007	89.1	14,042	75.1	14,042	48.0	14,042
Indonesia-2012	93.3	14,782	80.4	14,782	64.4	14,782
Philippines-2003	83.8	4,802	53.2	4,802	40.1	4,802
Philippines-2008	87.7	4,590	54.0	4,590	46.7	4,590

### Timing of the first antenatal care visit

Table 2 shows that in Indonesia (2012), among the women more than three ANC visits, more than 80% received it in the first trimester, which is highest among the selected countries. Among the women had more than three ANC visits, more than 50% received it in the first trimester in the Philippines (2008) whereas this percentage is lowest for Bangladesh. The DHS survey conducted in Bangladesh in the year 2004 and 2007 shows that the percentage of women going for ANC visits in the first trimester were only 19% and 24% respectively. But it is notable that this percentage has increased over the period in all the selected countries.

### Place of delivery

Table 2 shows that Bangladesh has the lowest percentage of institutional delivery with only 10% and 16% of the women going for institutional delivery in the year 2004 and 2007 respectively. The DHS data for Nepal shows though there has been a two-fold increase in institutional delivery from 2006 to 2011, still home delivery remains high at 62%. The DHS survey for Pakistan shows that the percentage of institutional delivery has increased from 37% in 2006–07 to 51% in 2012–13. India marks a relatively slow progress with an increase of only eight percent in the utilization of institutional delivery from NFHS (1998–99) to (2005–06).

### Probit and biprobit analysis

Tables 3 and 4 shows that the percentages of institutional delivery by the two dimensions of ANC while Tables 5 and 6 shows the estimated coefficients from the probit and biprobit model for institutional delivery. The analysis shows that both the dimensions of ANC show a positive effect on institutional delivery in the single-equation probit model as well as in the biprobit model. In the biprobit model, the rho association tells about the endogeneity in the system of equations. This means if there is a association between the two errors in the biprobit equation then the estimates of probit model gives a biased estimate of the coefficients and hence there is the need for the biprobit model to be employed to get a correct estimate of the true association. The level of association between the two dimensions of ANC and institutional delivery varies in the study countries. The endogenous nature of the explanatory variables are different in the study countries and is also absent in some countries. The country wise results are presented below.

**Table 3:** Percentages of institutional delivery according to the dimensions of ANC.

Country, Year, Dimensions of ANC	Factor	Place of delivery (%)		Total
		Home	Institutional	
**Bangladesh: 2004**	***No of ANC visits***			** **
**Independent of Health Care Delivery Systems**	<3 visits®	95.2	4.8	3,775
** **	3+ visits	72.6	27.4	1,591
**Depends on Health Care Delivery Systems**	***ANC care***			
	Level-1,2 = 0®	96.5	3.5	3,678
	Level-3 = 1	70.9	29.1	1,688
**Bangladesh: 2007**	***No of ANC visits***			
**Independent of Health Care Delivery Systems**	<3 visits®	93.7	6.3	3,238
** **	3+ visits	61.7	38.3	1,688
**Depends on Health Care Delivery Systems**	***ANC care***			
	Level-1,2 = 0®	91.0	9.0	4,145
	Level-3 = 1	38.0	62.0	781
**Pakistan: 2006–07**	***No of ANC visits***			
**Independent of Health Care Delivery Systems**	<3 visits®	79.9	20.1	3,481
** **	3+ visits	37.7	62.3	2,243
**Depends on Health Care Delivery Systems**	***ANC care***			
	Level-1,2 = 0®	74.1	25.9	4,511
	Level-3 = 1	24.5	75.5	1,213
**Pakistan: 2012–13**	***No of ANC visits***			
**Independent of Health Care Delivery Systems**	<3 visits®	68.9	31.1	3,654
** **	3+ visits	27.1	72.9	3,807
**Depends on Health Care Delivery Systems**	***ANC care index***			
	Level-1 = 0®	69.0	31.0	3,884
	Level-2 = 1	22.8	77.2	3,577
**Nepal: 2006**	***No of ANC visits***			
**Independent of Health Care Delivery Systems**	<3 visits®	93.5	6.5	2,174
** **	3+ visits	67.3	32.7	2,008
**Depends on Health Care Delivery Systems**	***ANC care***			
	Level-1,2 = 0®	90.8	9.2	3,201
	Level-3 = 1	47.7	52.3	981
**Nepal: 2011**	***No of ANC visits***			
**Independent of Health Care Delivery Systems**	<3 visits®	86.0	14.0	1,271
** **	3+ visits	49.1	50.9	2,808
**Depends on Health Care Delivery Systems**	***ANC care***			
	Level-1,2 = 0®	77.3	22.7	2,570
	Level-3 = 1	35.2	64.8	1,509
**India: 1998–99**	***No of ANC visits***			
**Independent of Health Care Delivery Systems**	<3 visits®	86.3	13.7	15,683
** **	3+ visits	40.6	59.4	13,295
**Depends on Health Care Delivery Systems**	***ANC care***			
	Level-1,2 = 0®	78.2	21.8	22,800
	Level-3 = 1	22.9	70.1	6,178

**Table 4:** Percentages of institutional delivery according to the dimensions of ANC.

Country, Year, Dimensions of ANC	Factor	Place of delivery (%)		Total
		Home	Institutional	
**India: 2005–06**	***No of ANC visits***			
**Independent of Health Care Delivery Systems**	<3 visits®	83.0	17.0	14,457
** **	3+ visits	36.3	63.7	22,393
**Depends on Health Care Delivery Systems**	***ANC care***			
	Level-1 = 0®	71.0	29.0	15,103
	Level-2 = 1	20.4	79.6	14,931
**Indonesia: 2007**	***No of ANC visits***			
**Independent of Health Care Delivery Systems**	<3 visits®	83.3	16.7	2,108
** **	3+ visits	48.2	52.8	13,266
**Depends on Health Care Delivery Systems**	***ANC care***			
	Level-1,2 = 0®	60.3	39.7	11,162
	Level-3 = 1	32.1	67.9	4,172
**Indonesia: 2012**	***No of ANC visits***			
**Independent of Health Care Delivery Systems**	<3 visits®	80.4	19.6	1,441
** **	3+ visits	32.4	67.6	13,821
**Depends on Health Care Delivery Systems**	***ANC care***			
	Level-1,2 = 0®	42.1	57.9	10,445
	Level-3 = 1	23.8	76.2	4,817
**Philippines: 2003**	***No of ANC visits***			
**Independent of Health Care Delivery Systems**	<3 visits®	84.4	15.6	823
** **	3+ visits	55.1	48.9	4,097
**Depends on Health Care Delivery Systems**	***ANC care***			
	Level-1,2 = 0®	71.4	28.6	3,489
	Level-3 = 1	33.8	66.2	1,431
**Philippines: 2008**	***No of ANC visits***			
**Independent of Health Care Delivery Systems**	<3 visits®	79.3	20.7	624
** **	3+ visits	49.6	50.4	4,088
**Depends on Health Care Delivery Systems**	***ANC care***			
	Level-1,2 = 0®	65.6	34.4	3,206
	Level-3 = 1	30.4	69.6	1,506

® denotes the reference category

**Table 5:** Probit and biprobit estimation for institutional delivery.

		Probit model		Biprobit model		
Institutional delivery	Factor	Coef.	Predicted probability	Coef.	Predicted probability	Behaviour of rho
**Bangldesh-2004**	***No of ANC visits***					
**Independent of Health Care Delivery Systems**	<3 visits®		0.05		0.09	
** **	3+ visits	0.55**	0.28	0.53**	0.18	rho = 0
**Depends on Health Care Delivery Systems**	***ANC care***					
	Level-1,2 = 0®		0.04		0.07	
	Level-3 = 1	0.76**	0.29	0.75**	0.20	rho = 0
**Bangldesh-2007**	***No of ANC visits***					
**Independent of Health Care Delivery Systems**	<3 visits®		0.06		0.17	rho<0
** **	3+ visits	0.73**	0.38	1.09**	0.25	-0.259
**Depends on Health Care Delivery Systems**	***ANC care index***					
	Level-1,2 = 0®		0.09		0.14	rho<0
	Level-3 = 1	1.00**	0.62	1.69**	0.32	-0.424
**Pakistan-2006-07**	***No of ANC visits***					
**Independent of Health Care Delivery Systems**	<3 visits®		0.21		0.27	rho<0
** **	3+ visits	0.74**	0.62	0.92**	0.50	-0.129
**Depends on Health Care Delivery Systems**	***ANC care***					
	Level-1,2 = 0®		0.26		0.31	rho<0
	Level-3 = 1	0.78**	0.74	1.49**	0.53	-0.44
**Pakistan-2012-13**	***No of ANC visits***					
**Independent of Health Care Delivery Systems**	<3 visits®		0.29		0.42	rho<0
** **	3+ visits	0.84**	0.75	1.16**	0.66	-0.246
**Depends on Health Care Delivery Systems**	***ANC care index***					
	Level-1 = 0®		0.30		0.41	rho<0
	Level-2 = 1	0.89**	0.78	1.47**	0.68	-0.402
**Nepal-2006**	***No of ANC visits***					
**Independent of Health Care Delivery Systems**	<3 visits®		0.06		0.14	rho<0
** **	3+ visits	0.6**	0.31	1.24**	0.22	-0.437
**Depends on Health Care Delivery Systems**	***ANC care index***					
	Level-1,2 = 0®		0.08		0.14	rho<0
	Level-3 = 1	0.72**	0.51	1.38**	0.27	-0.409
**Nepal-2011**	***No of ANC visits***					
**Independent of Health Care Delivery Systems**	<3 visits®		0.14		0.29	rho<0
** **	3+ visits	0.67**	0.51	1.06**	0.44	-0.277
**Depends on Health Care Delivery Systems**	***ANC care index***					
	Level-1,2 = 0®		0.23		0.33	rho<0
	Level-3 = 1	0.63**	0.66	1.35**	0.50	-0.457

Significance at

* p<0.05

** p<0.01

*** p<0.001

® denotes the reference category

**Table 6:** Probit and biprobit estimation for institutional delivery.

Institutional delivery	Factor	Probit model Coef.	Predicted probability	Biprobit model Coef.	Predicted probability	Behaviour of rho
**India-1998-99**	***No of ANC visits***					
**Independent of Health Care Delivery Systems**	<3 visits®		0.13		0.25	rho<0
** **	3+ visits	0.82**	0.58	1.09**	0.46	-0.193
**Depends on Health Care Delivery Systems**	***ANC care index***					
	Level-1,2 = 0®		0.21		0.30	rho<0
	Level-3 = 1	0.88**	0.76	1.40**	0.52	-0.322
**India-2005-06**	***No of ANC visits***					
**Independent of Health Care Delivery Systems**	<3 visits®		0.16		0.35	rho<0
** **	3+ visits	0.81**	0.63	1.07**	0.56	-0.187
**Depends on Health Care Delivery Systems**	***ANC care index***					
	Level-1 = 0®		0.28		0.40	rho<0
	Level-2 = 1	0.88**	0.79	1.48**	0.66	-0.385
**Indonesia-2007**	***No of ANC visits***					
**Independent of Health Care Delivery Systems**	<3 visits®		0.14		0.36	rho<0
** **	3+ visits	0.46**	0.48	0.83**	0.43	-0.252
**Depends on Health Care Delivery Systems**	***ANC care***					
	Level-1,2 = 0®		0.36		0.38	rho<0
	Level-3 = 1	0.41**	0.64	1.16**	0.49	-0.465
**Indonesia-2012**	***No of ANC visits***					
**Independent of Health Care Delivery Systems**	<3 visits®		0.19		0.42	rho<0
** **	3+ visits	0.72**	0.64	1.08**	0.57	-0.239
**Depends on Health Care Delivery Systems**	***ANC care***					
	Level -1,2 = 0®		0.53		0.52	rho<0
	Level -3 = 1	0.40**	0.75	1.12**	0.63	-0.46
**Philippines-2003**	***No of ANC visits***					
**Independent of Health Care Delivery Systems**	<3 visits®		0.16		0.29	
** **	3+ visits	0.40**	0.44	0.56**	0.39	rho = 0
**Depends on Health Care Delivery Systems**	***ANC care***					
	Level -1,2 = 0®		0.28		0.33	
	Level -3 = 1	0.49**	0.65	0.71**	0.47	rho = 0
**Philippines-2008**	***No of ANC visits***					
**Independent of Health Care Delivery Systems**	<3 visits®		0.21		0.37	
** **	3+ visits	0.38**	0.51	0.67**	0.45	rho = 0
**Depends on Health Care Delivery Systems**	***ANC care***					
	Level -1,2 = 0®		0.34		0.39	
	Level -3 = 1	0.52**	0.71	0.82**	0.54	rho = 0

Significance at

* p<0.05

** p<0.01

*** p<0.001

® denotes the reference category

### Bangladesh: Bangladesh Demographic Health Survey, 2004 and 2007

The Bangladesh Demographic and Health Survey (BDHS)-2004 data shows that the institutional delivery increases from 5% when the women had less than three ANC visits to 27% when women had more than three ANC visits. Similarly for BDHS-2007 the institutional delivery increases from 4% when the women had less than three ANC visits to 29% when women have more than three ANC visits. On a similar line, the higher level of ANC care led to 33% (29% to 62%) increase in the institutional deliveries between 2004 to 2007 (Table 3).

Tables 5 and 6 shows that both the dimensions of ANC are positively related to the utilization of institution delivery. The probit model suggests that women completing more than three ANC visits are more likely to go for institutional delivery than the reference category (less than three visits). The predicted probabilities for the utilization of institutional delivery are 0.05 and 0.28 when the dimension of ANC is independent of the health care delivery systems, and the corresponding values are 0.04 and 0.29 when the dimension of ANC is dependent on health care delivery systems. This suggests that when women utilize three or more ANC visits, they are more likely to go for institutional delivery as compared to those women who have less than three ANC visits. On the other hand, women have 25 percent higher chance to go for institutional delivery when the level of ANC care is high. The same model gives a higher association between the two dimensions of ANC and institutional delivery for BDHS-2007.

The biprobit estimation has been employed to look at the endogenous nature of the key explanatory variables. The biprobit estimation of BDHS-2004 shows that both the dimensions of ANC care are exogenous in nature because the error association has been found as zero using debut of “first antenatal care in first trimester” variable as the instrument. However, the presence of endogeneity is found while estimating the association for BDHS-2007 data. The negative association is 26% between the two errors in the biprobit equation for the first dimension, and it is 42% for the second dimension. The biprobit estimation for BDHS-2007 data shows that the probability of going for institutional delivery is eight percent more when women utilize three or more ANC visits as compared to less than three ANC visits, while it is 18% more when the level of ANC care is high compared to the low level of ANC care. This indicates that dimension of ANC which depends on the health care delivery systems is having a greater influence on institutional delivery as compared to the dimension of ANC which is independent of health care delivery systems.

### Pakistan: Pakistan Demographic Health Survey, 2006–07, and 2012–13

The Pakistan Demographic and Health Survey (PDHS)-2006-07 data shows that 62% of the women who had completed three or more ANC visits have gone for institutional delivery, and this percentage has increased to 73% for PDHS-2012-13. If the women are at the high level of ANC care, then the percentage of institutional delivery are 76% and 77% for PDHS-2006-07 and 2012–13 respectively (Table 3). Table 5 shows that both the dimensions of ANC hold a positive relationship with institutional delivery. Probit model suggests that both the dimensions of ANC are highly significant for institutional delivery. In the biprobit estimation, endogeneity is present for both the dimensions and the associations are negative with 13% and 44% for PDHS 2006–07 and 25% and 40% for PDHS 2012–13 for the first and second dimension of ANC respectively. This reflects that some unobserved factors are affecting the estimates which show the influence of the two dimensions of ANC on institutional delivery. After employing biprobit estimation, it is found that those women who had gone more than three ANC visits have 23% more chance to go for institutional delivery as compared to women with less than three ANC visits. On the other hand, the chance to go for institutional delivery is 22% more for those women who have received the high level of ANC care as compared to those who received low level of ANC care. Similarly, the PDHS-2012-13 dataset shows that the estimated coefficients are improving from probit to biprobit estimation. Women who had gone more than three ANC visits in 2012–13 have 24% more chance to go for institutional delivery as compared to women with less than three ANC visits. Moreover, the chance to go for institutional delivery is 27% more for those women who have received the high level of ANC care than those who had received low level of ANC care.

### Nepal: Nepal Demographic Health Survey -2006 and 2011

The Nepal Demographic and Health Survey (NDHS) data shows that the percentage of three or more ANC visits are 49% and 67% and the coverage of institutional deliveries are 19% and 39% for 2006 and 2011 respectively (Table 2). The NDHS-2006 data shows that the institutional delivery is 33% among the women who had gone for more than three ANC visits while it is only 7% among women who had gone for less than three ANC visits. On a similar line, the NDHS-2011 data shows that the institutional delivery is 51% among women who had gone for more than three ANC visits while it is only 14% among women who had gone for less than three ANC visits. The NDHS-2006 data shows that 52% of the women who had received the high level of ANC care have gone for institutional delivery while this figure has increased to 65% for NDHS-2011 (Table 3).

It is found that after biprobit estimation the estimates are improving from 0.60 to 1.24 for the first dimension of ANC and from 0.72 to 1.38 for the second dimension of ANC. NDHS-2006 data shows that the probability of institutional delivery is eight percent more when women utilize three or more ANC visits as compared to less than three ANC visits, while the institutional delivery is 13% more when the level of ANC care is high as compared to low level of ANC care. During NDHS-2011 the probability of institutional delivery for the first and second dimensions of ANC was 15% and 17% respectively. This reflects the dimension of ANC which depends on health care delivery systems is having a greater influence on institutional delivery as compared to the dimension of ANC which is independent of the health care delivery systems.

### India: National Family Health Survey 1998–99 and 2005–06

The NFHS-1998-99 data for India shows that 59% of women who had completed three or more ANC visits have gone for institutional delivery, and this percentage has slightly increased and reached 64% in NFHS-2005-06. If the women are receiving high level of ANC care, then the percentage of institutional delivery are 70% and 80% for NFHS-1998-99 and NFHS-2005-06, respectively (Table 3). The probit coefficients infer that the second dimension of ANC is more intense to influence mothers for the uptake of institutional delivery. It is also observed that the error associations are more in the second dimension of ANC compared to the first dimension. This indicates that the unobservable factors are more associated with the second dimension making this key factor more endogenous in nature and at the same time if endogeneity is corrected then this factor will have a stronger influence on the mothers for the uptake of institutional delivery. The probabilities obtained through biprobit estimation for the dimension of ANC independent of the health care system shows that if women have more than three ANC visits the probability of institutional delivery is 0.46% for NFHS-1998-99 and 0.56% for NFHS-2005-06 thus reflecting an improvement of 10 points in probability. For the ANC dimension dependent on the health care delivery systems the probability of institutional delivery is 0.52% and 0.66% in NFHS-1998-99 and NFHS-2005-06, respectively (Table 6).

### Indonesia: Indonesia Demographic and Health Survey, 2007 and 2012

According to the Indonesia Demographic and Health Survey (IDHS)-2007, 4,172 women received the highest level of ANC care and among them 68% utilized institutional delivery, while in IDHS-2012, out of 4,817 women who received the highest level of ANC care 76% utilized institutional delivery (Table 4). Probit and biprobit estimations also show a similar pattern of association between the two dimensions of ANC and institutional delivery. The rho associations are also found negative for Indonesia. Biprobit estimation for IDHS-2007 reflects that the women who had more than three ANC visits have seven percent higher probability of having institutional delivery as compared to the women who had less than three ANC visits. On the other hand, the probability to have institutional delivery is 11% more for women who are exposed to the highest level of ANC care as compared to their counterparts. The IDHS-2012 also shows similar patterns (Table 6). Thus it is found that the second dimension of ANC remained more effective for the promotion of institutional delivery among mothers in Indonesia.

### Philippines: The Philippines National Demographic and Health Survey, 2003 and 2008

The two consecutive rounds of the Philippines National Demographic and Health Survey (NDHS)-2003 and 2008 show that among the women who received the highest level of ANC care, 66% and 70% respectively have gone for institutional delivery (Table 4). For the Philippines, both the dimensions of ANC behaves as an exogenous variable and thus after employing the biprobit model it was found that the coefficients have not improved much as in the other study countries (Table 6). According to the NDHS-2003 data, the probability to deliver in an institution is 0.44 if women have more than 3 ANC visits while the probability is 0.16 if the women have made less than three ANC visits. The same estimation gives a probability of 0.28 and 0.65 for low level and high level of ANC care respectively. According to the NDHS-2008 data, the probability of having institutional delivery is 0.21 when women have more than three ANC visits while the probability increases to 0.71 when the women have received high level of ANC care. So, for the Philippines also it was found that the dimension of ANC not dependent on the health care delivery systems is more associated with the utilization of institutional delivery as compared to the first dimension of ANC.

## Discussion and conclusions

Previous studies have tried to determine the association between ANC utilization and institutional delivery by considering the number of ANC visits, and it is well known that only ANC visits cannot be the totality of the ANC process. Therefore, this study is a break point where ANC is considered in two separate dimensions, utilizing the full information on ANC which is available in the DHS. This study investigates the impact of ANC when ANC components are dependent or independent from health care delivery systems on institutional delivery in the selected countries while attempting to correct the problems of endogeneity to capture the true association. The study further tries to compare the two dimensions to check which one is more intensely associated with institutional delivery. The study helps in providing a better understanding of the facts and insights of ANC and institutional delivery interlinkages and the promotional aspects of ANC for institutional delivery among the mothers.

The inference of this study is based on the DHS data for six selected South and South East Asian countries. Looking at the purpose of the study sophisticated statistical tools have been employed for analyzing the data. The study primarily examines the bivariate distributions and then assesses the associations empirically. The probit estimation method has been applied assuming the two key predictor variables being exogeneous. At the next step, the analysis has been taken to the advanced level where biprobit estimation is introduced to nullify the endogeneity problem while predicting the true associations between the two dimensions of ANC and institutional delivery.

Giving birth in health facilities has a strong positive influence on the health of mothers and their children, and there is a growing trend for women to give birth in health facilities globally both in private and public health institutions [15]. Among the study countries, Indonesia shows a high percentage of births in health facilities. Otherwise, in most of the study countries, the majority of women delivered in a non-institutional setting.

Two different probit models have been estimated to check the two factors’ association in two separate models assuming exogeneity. The estimated coefficients are then tested at five percent level of significance. Next, a biprobit model has been employed for each of the factors to improve further the estimates correcting the endogeneity bias, parallel to this estimation Wald test of exogeneity is performed to check the associations between the two errors, i.e. the rho value in the biprobit equation. These results are compared with those estimated from probit models to assess the likely effects of biases when endogeneity issues are not taken into account. Comparison of probit and biprobit estimates shows that except Bangladesh (BDHS-2004) and Philippines (NDHS-2003 and 2008) in other countries, unobserved factors underestimate the positive impact of both the dimensions of ANC on Institutional delivery. The test ensured that coefficient of the ‘second dimension of ANC’ which depends on health care delivery systems are always greater than the counterpart in all the study countries.This suggests that ANC components which depend on health care delivery systems are more strongly associated with the utilization of institutional delivery. Further, it shows that just visiting health center during pregnancy does not ensure that institutions provide services efficiently. It also does not ensure that appropriate ANC is arranged and, therefore, decent quality and quantity of information with education are delivered to motivate women for uptake of next level of services.

The results of this study are relevant to maternal health policies and programs in the least developed countries of the world, where maternal health services remain relatively less used, and the importance of antenatal services is under debate [29]. The study also highlights the importance of improving the quality of ANC which depends on health care delivery systems as it would promote the utilization of institutional delivery. The content of care may fulfill the women’s need and expectations while visiting for ANC care. Therefore, it motivates the women to utilize the institutional delivery. Studies carried out in various countries demonstrate that women are still not being systematically given adequate information during the time of ANC visits. The service providers who do so typically only cite the benefits of the skilled birth attendant and often make little attempt to understand the cultural practices and the social norms of the area and address to women accordingly [29]. Therefore, the programs and policies should intervene in a way to promote ANC care utilization in the best possible way, and better quality of care should be provided by healthcare workers keeping in mind the women’s perspectives. At the same time, the government should improve the healthcare infrastructure and increase the number of health personnel to meet the health care needs.

## Ethics statement

This study used datasets from demographic health surveys which are a publicly available dataset and with no access to personal identifiers.
